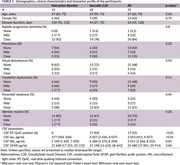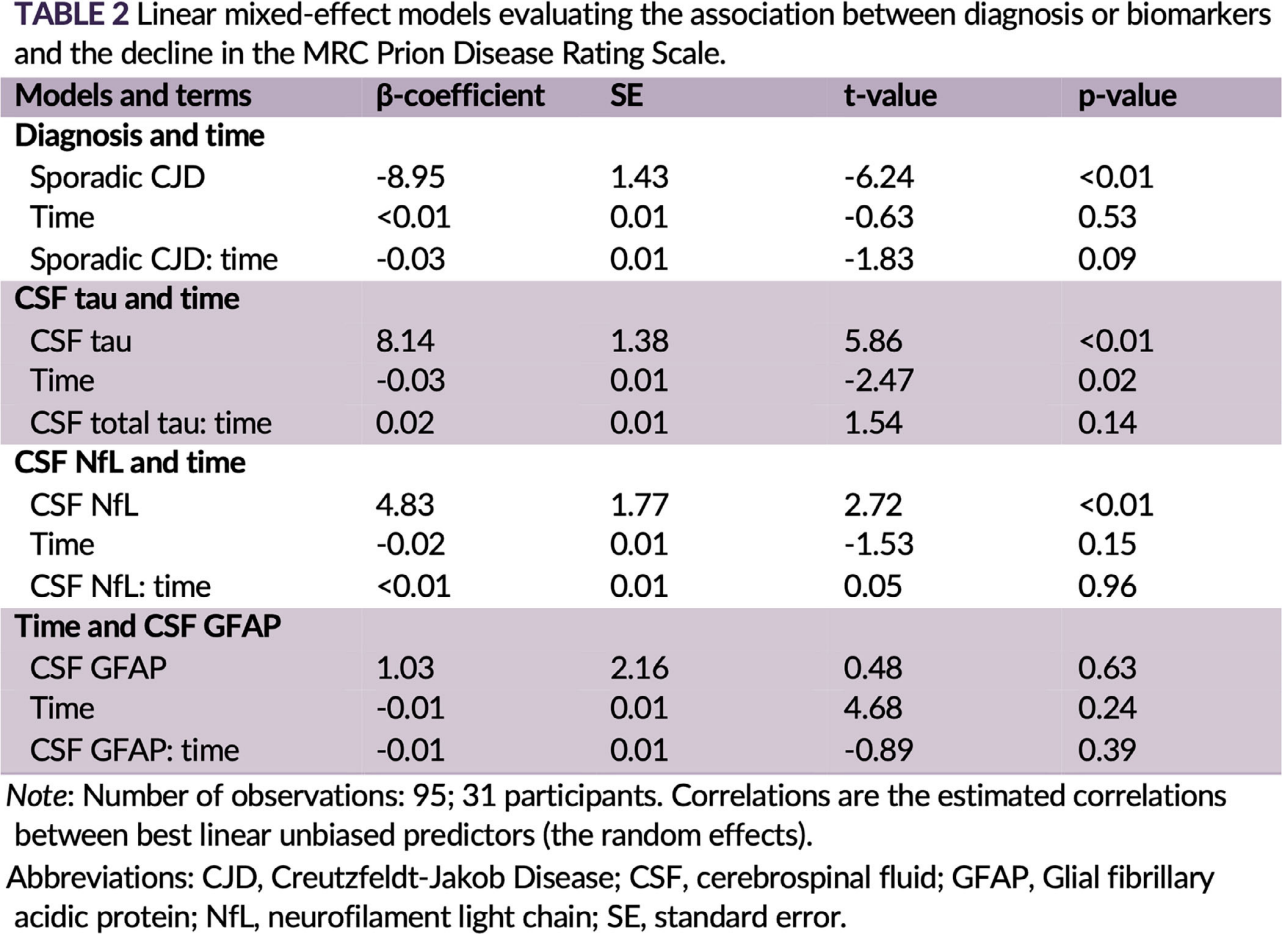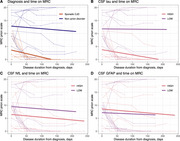# Predictors of Disease Progression Among Patients in The Thai National Prion Disease Surveillance Program

**DOI:** 10.1002/alz70856_104662

**Published:** 2026-01-07

**Authors:** Chayanis Yolsiriwat, Adipa Chongsuksantikul, Prawit Oangkhana, Watayuth Luechaipanit, Thanaporn Haethaisong, Abhinbhen Wasontiwong Saraya, Poosanu Thanapornsangsuth

**Affiliations:** ^1^ King Chulalongkorn Memorial Hospital, Bangkok, Bangkok, Thailand; ^2^ Thai Red Cross Emerging Infectious Diseases Health Science Centre, King Chulalongkorn Memorial Hospital, Bangkok, Thailand; ^3^ Thai Red Cross Emerging Infectious Diseases Health Science Centre, King Chulalongkorn Memorial Hospital, The Thai Red Cross Society, Bangkok, Thailand; ^4^ King Chulalongkorn Memorial Hospital, Bangkok, Thailand; ^5^ Thai Red Cross Emerging Infectious Diseases Health Science Centre, World Health Organization Collaborating Centre for Research and Training on Viral Zoonoses, King Chulalongkorn Memorial Hospital, The Thai Red Cross Society, Bangkok, Thailand; ^6^ Memory Clinic, King Chulalongkorn Memorial Hospital, The Thai Red Cross Society, Bangkok, Thailand

## Abstract

**Background:**

The unfavorable prognosis of prion diseases highlights the need for a robust diagnostic approach system to accurately distinguish them from other etiologies of dementia. Correlating biomarkers is crucial for advancing therapies and optimizing patient selection in clinical trials.

**Method:**

We prospectively enrolled participants with suspected prion disease from hospitals across Thailand from March 2023 to December 2024. Standardized questionnaires were developed to collect clinical and demographic data, disease‐specific exposures, and laboratory findings, including blood and CSF tests, brain MRI, and EEG. CSF RT‐QuIC was performed in all cases, with NfL and GFAP measured using the SIMOA™ assay (Quanterix) and tau levels determined via ELISA (EUROIMMUN). Disease progression is monitored using the MRC Prion Disease Rating Scale. Baseline characteristics and investigational biomarkers were compared between sporadic CJD and non‐prion disorders. Linear mixed‐effect models were used to evaluated interactions between each biomarker and time, as these interactions directly represent disease progression. Predictive biomarkers were dichotomized using median levels as cut‐offs to enable clearer group comparisons.

**Result:**

Among 31 patients, 42% were female with mean age of 67 years. The most common clinical presentation is rapidly progressive dementia (84%) followed by pyramidal weakness, myoclonus and cerebellar dysfunction. There are no significant differences between core clinical features between both groups. CSF RT‐QuIC was tested positive in almost all sporadic CJD and non in non‐prion disorders. CSF tau is higher in sporadic CJD (4,027 pg/mL vs 377 ng/mL, *p*‐value<0.001). (Table 1) Sporadic CJD diagnosis and higher levels of CSF tau and CSF NfL reported a worse MRC score at baseline. (Figure 2A‐C) Linear mixed‐effects models revealed no significant interactions between CSF biomarkers and time to MRC score. (Table 2)

**Conclusion:**

Our study demonstrates a significant main effect of CSF tau and NfL on the MRC score but no interaction effects. Patients with higher CSF tau levels generally have worse functional status throughout the clinical course, with similar rates of decline. However, the limited power of this study may restrict the detection of interaction effects.